# Target Detection Method for Soil-Dwelling Termite Damage Based on MCD-YOLOv8

**DOI:** 10.3390/s25072199

**Published:** 2025-03-31

**Authors:** Peidong Jiang, Lai Jiang, Fengyan Wu, Tengteng Che, Ming Wang, Chuandong Zheng

**Affiliations:** 1 Hubei Water Resources Research Institute, Wuhan 430070, China; 13607266521@163.com (P.J.); wufengyan1981@126.com (F.W.); xiaoxuan0703@163.com (T.C.); wang130ming1@gmail.com (M.W.); 2 Key Laboratory of Termite Control of Ministry of Water Resources, Hubei Provincial Department of Water Resources, Wuhan 430071, China; 3 Yangxin County Bureau of Water Resources and Lakes, Huangshi 435200, China; m15623522653@163.com

**Keywords:** soil-dwelling termite damage, image recognition, light weight, improved YOLOv8 model

## Abstract

With global climate change and the deterioration of the ecological environment, the safety of hydraulic engineering faces severe challenges, among which soil-dwelling termite damage has become an issue that cannot be ignored. Reservoirs and embankments in China, primarily composed of earth and rocks, are often affected by soil-dwelling termites, such as Odontotermes formosanus and Macrotermes barneyi. Identifying soil-dwelling termite damage is crucial for implementing monitoring, early warning, and control strategies. This study developed an improved YOLOv8 model, named MCD-YOLOv8, for identifying traces of soil-dwelling termite activity, based on the Monte Carlo random sampling algorithm and a lightweight module. The Monte Carlo attention (MCA) module was introduced in the backbone part to generate attention maps through random sampling pooling operations, addressing cross-scale issues and improving the recognition accuracy of small targets. A lightweight module, known as dimension-aware selective integration (DASI), was added in the neck part to reduce computation time and memory consumption, enhancing detection accuracy and speed. The model was verified using a dataset of 2096 images from the termite damage survey in hydraulic engineering within Hubei Province in 2024, along with images captured by drone. The results showed that the improved YOLOv8 model outperformed four traditional or enhanced models in terms of precision and mean average precision for detecting soil-dwelling termite damage, while also exhibiting fewer parameters, reduced redundancy in detection boxes, and improved accuracy in detecting small targets. Specifically, the MCD-YOLOv8 model achieved increases in precision and mean average precision of 6.4% and 2.4%, respectively, compared to the YOLOv8 model, while simultaneously reducing the number of parameters by 105,320. The developed model is suitable for the intelligent identification of termite damage in complex environments, thereby enhancing the intelligent monitoring of termite activity and providing strong technical support for the development of termite control technologies.

## 1. Introduction

Termites, as ancient social insects distributed nearly globally, play an essential role in the Earth’s ecosystem [1]. However, their small size and extensive populations contribute to the covert foraging behaviors that result in considerable economic damage, categorizing them as significant pests [2]. The intrusion of human activities into termite habitats, coupled with the effects of climate change, has intensified their impact, particularly in the Yangtze and Pearl River basins in China, where the damage caused by soil-dwelling termites is particularly pronounced [3]. In China, the primary areas affected by termite damage include hydraulic engineering [4,5,6], buildings [7,8], and landscaping [9], with annual economic losses estimated between 2 to 2.5 billion yuan [10]. The characteristics of termite underground activity complicates detection and control efforts. The damage inflicted by termites frequently demonstrates strong concealment and rapid spread [11,12]. Consequently, the termite control in hydraulic engineering has emerged as a central focus of preventive strategies in China. In 2023, the Ministry of Water Resources in China underscored principles, such as “technological innovation” and “green and safety”. The Ministry advocated for innovation-driven methodologies, enhanced scientific research on termite control, the accelerated adoption of cutting-edge technologies and equipment, and the encouraged use of environmentally sustainable practices in termite control.

Traditional methods for identifying termite damage rely on manual field surveys and visual observation to find the indicators of termite activity (such as mud tunnels, swarming holes, and plants that are eaten by moths), which are labor-intensive and inefficient [13]. Recently, with advancements in computer vision and deep learning, intelligent methods for identifying termite damage have emerged. Lopes et al. introduced convolutional neural networks (CNNs) to assess the severity of termite damage, employing data augmentation techniques to enhance the original dataset of 181 samples and evaluating the performance of four distinct models [14]. Their findings indicated that the AlexNet model exhibited commendable performance on this dataset, although the results may be influenced by the dataset’s limited size. In a separate study, Huang et al. developed a deep neural network model to intelligently identify termite species, achieving over 90% accuracy in identifying worker and soldier termites using 18,000 images of different species [15]. Additional researchers have investigated the potential of utilizing termite sounds [16], volatile pheromones [17], carbon dioxide concentrations [18], and infrared thermography [19] for identification purposes, but these methods are mostly in the experimental stage with limited practical applications. Overall, the current state of intelligent technologies for termite damage identification is nascent and presents several challenges. For instance, these technologies are infrequently employed in assessing termite risks in dam structures, and many are constrained by reliance on single-source data, inadequate data volume, and diverse identification targets [13]. This results in low integration and challenges in adapting to varying geographic and lighting conditions.

Additionally, soil-dwelling termite activity signs mainly include mud tunnels, mud coverings, and swarming holes constructed by termites using soil, showcasing unique morphological characteristics [13]. Indicators of soil-dwelling termite damage frequently manifest within intricate forested environments and are typically of a centimeter scale, categorizing them as small objects in the realm of image recognition. Currently, the accuracy of the YOLOv8 algorithm in detecting small objects requires enhancement, prompting various researchers to propose strategies for improvement [20,21,22,23]. For example, Dai proposed a scale-variant attention network (SvANet) for the precise segmentation of small objects in medical images [24]. Xu et al. developed a deep learning framework known as HCF-Net, which incorporates multiple practical modules to significantly advance infrared small object detection, effectively addressing challenges associated with the diminutive size of targets and the complexity of infrared image backgrounds [25]. To improve the detection of the indicators of termite damage and enhance accuracy for small objects, it is necessary to develop an image recognition model specifically designed to accommodate the unique characteristics of soil-dwelling termite activity signs.

Current methods often lack the necessary adaptability and precision for such niche scenarios, underscoring the need for a more tailored approach. Our study fills this gap by introducing MCD-YOLOv8, a model specifically designed to address the unique challenges of small target detection in soil-dwelling termite activity signs. The main innovations of this study are as follows:

(1) The development of a termite damage feature dataset tailored for multi-target detection, which encompasses four distinct labels: deadwood, detector, hole, and mud;
(2) The MCA module is introduced in the backbone part, improving the accuracy of small object detection. This module extracts local and contextual information through random sampling, thereby increasing the recognizability of small objects;
(3) The DASI module is added in the neck part to reduce computation time and memory consumption, enhancing detection accuracy and speed, enabling effective multi-scale feature fusion.

This research contributes to the timely detection and localization of termite-related damage, thereby mitigating potential damage losses, and holds considerable theoretical and practical significance for improving the level of termite control technologies.

## 2. Related Work

### 2.1. Target Detection Methods

In recent years, artificial intelligence technology has advanced rapidly, with algorithms based on deep learning theory achieving significant breakthroughs in the field of target detection. These algorithms are mainly categorized into two types [26]: two-stage algorithm and one-stage algorithm. Two-stage algorithms, like R-CNN [27] and FPN [28], generate candidate regions before conducting classification and bounding box regression. Although this method achieves high precision, it is computationally complex and has limited real-time performance [26]. On the other hand, single-stage algorithms, like SSD [29] and the YOLO series [30], bypass the candidate region generation, allowing for quick classification and localization, making them suitable for complex scenarios requiring instant response. Despite the accuracy advantage of the two-stage algorithm and the one-stage algorithm, especially the YOLO series, have demonstrated exceptional detection accuracy and efficient processing capabilities across numerous applications, particularly in the real-time detection of soil-dwelling termite activity traces in complex environments.

Serving as a paradigm of single-stage algorithms, YOLO excels in real-time applications with its outstanding detection speed and accuracy and is widely used in fields such as medicine, transportation, and agriculture [31]. Gu et al. innovatively introduced the YOLOv8-Skin, incorporating multi-scale features and edge enhancement, significantly improving the accuracy of melanoma segmentation [32]. Zhang et al. developed the RBT-YOLO, optimizing vehicle detection precision through multi-scale fusion and triplet attention, thus markedly enhancing performance while reducing computational demands [33]. Wang et al. optimized YOLOv8 using an adaptive color perception module, enhancing feature extraction capabilities in complex natural environments [13]. However, despite the YOLO model demonstrating exceptional performance across various fields, challenges remain in adapting to various scenarios and accurately locating small targets.

### 2.2. Small Object Detection

Small object detection, as a subfield of object detection, faces significant technical challenges. In recent years, YOLOv8 has made notable progress in this field. To address the issues of low accuracy and susceptibility to environmental factors in small object detection, Wang et al. employed WIoU regression loss and gradient allocation to enhance the model’s feature extraction capabilities [34]. Jiang et al. proposed the AEM-YOLOv8s algorithm by combining the strengths of alterable kernel convolution and efficient multi-scale attention within the C2f module and incorporating a dedicated small object detection layer and BiFPN structure, which significantly improves the performance of small object detection [35]. Li et al. tackled the difficulties in feature extraction in complex scenes and the issue of small object features being easily obscured by noise by proposing the self position module (SPM) attention mechanism, which enhanced detection accuracy [36]. Currently, research mainly focuses on improving the perception of small objects [37], using methods such as multi-scale feature fusion, feature pyramid networks, and attention mechanisms. These studies provide effective technical support for detecting traces of soil-dwelling termite activity, but further improving the accuracy of small object detection in complex backgrounds and varying lighting conditions remains a key challenge.

Despite these advancements, current methods still struggle with detecting small targets, such as soil-dwelling termite activity, particularly under complex backgrounds and varying lighting conditions. The existing algorithms often fail to balance accuracy and computational efficiency in such challenging scenarios, highlighting the need for more robust and specialized models. To address these challenges, we propose an improved YOLOv8 model (MCD-YOLOv8) that leverages multi-scale feature fusion and an adaptive attention mechanism. Unlike previous models, our approach specifically optimizes for small target detection in complex environments by enhancing feature extraction capabilities and improving robustness against background noise and lighting variations.

## 3. Data Collection and Processing

### 3.1. Data Sources

**Figure 1:**
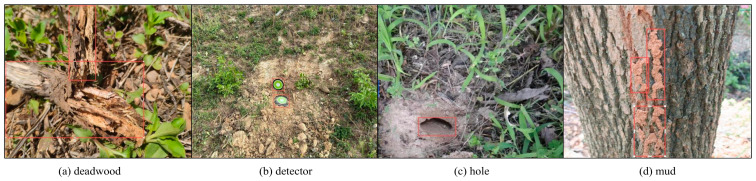
The image dataset of soil-dwelling termite damage. The red square frame represents reference examples for each category.

The dataset utilized in this study predominantly comprises images from termite damage surveys conducted in hydraulic engineering projects within Hubei Province in 2023, as well as images captured by a DJI Magic 3E drone (Shenzhen Dajiang Innovation Technology Co., Ltd., Shenzhen, China). It encompasses the following four distinct categories: deadwood, detector, hole, and mud, as illustrated in Figure 1. The survey images were acquired by technical personnel related to termite control in and around reservoirs and embankments, employing various angles and lighting conditions, which resulted in complex scenes characterized by small targets and multiple obstructions. The drone images ware captured along the dam axis directed towards the downstream slope, with parameters such as flight altitude and camera tilt angle in accordance with the height and slope of the dam. A total of 1093 raw images were compiled, including 196 drone images. All images within the dataset were meticulously annotated using Labelme v5.6.0 software and subsequently divided into training, validation, and testing sets in a ratio of 7:2:1. In contrast to indoor experimental data, this research leveraged thousands of preprocessed field images depicting soil-dwelling termite damage, thereby enhancing both the quantity and quality of samples, as well as improving the representativeness and diversity of the training set.

### 3.2. Data Augmentation

To reduce the risk of model overfitting and enhance the diversity and generalization ability of the training data, image augmentation techniques, such as mirroring, rotation, color transformation, and brightness adjustment, were applied to the original data [38,39,40]. These techniques replicate a range of lighting conditions and perspectives typically encountered in natural environments, thereby expanding the model’s applicability [41,42,43,44,45]. After data augmentation, a comprehensive dataset comprising 2096 images was generated. This enhancement effectively improves the model’s robustness, equipping it to effectively navigate the diverse and intricate natural environments. By introducing more environmental factors during model training, this approach not only increases the informational content of the training set but also enhances the model’s adaptability and stability in practical applications.

## 4. Improved YOLOv8 Model

### 4.1. YOLOv8 Model

**Figure 2:**
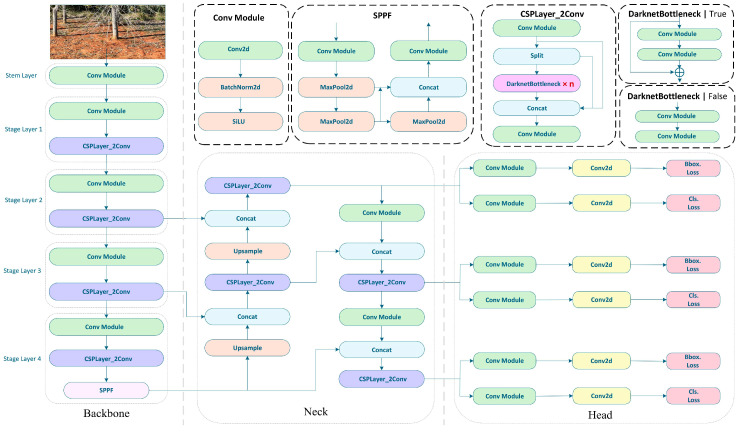
YOLOv8 model architecture.

As shown in Figure 2, the architecture of the YOLOv8 network is an evolution of the YOLOv5 framework, comprising three fundamental components: the backbone for feature extraction, the neck for feature fusion, and the head for final recognition and detection [46,47]. In the backbone, the C3 module from YOLOv5 is replaced with the C2f module [48] to achieve further lightweighting of the model. The architecture retains the spatial pyramid pooling (SPPF) module used in YOLOv5. The implementation of the C2f module in place of the C3 module contributes to the model’s lightweight characteristics. In contrast to YOLOv5, the neck of YOLOv8 eliminates the convolutional operations during the upsampling phase and replaces the C3 module with the more efficient C2f module, thereby enhancing the model’s adaptability to objects of varying sizes and shapes. The detection head adopts a decoupled structure, separating classification and detection, which results in a reduction in both parameter count and computational complexity, while simultaneously improving generalization and stability. YOLOv8 computes the bounding box loss via the Conv2d layer and departs from traditional anchor-based prediction methodologies in favor of an anchor-free approach, which directly predicts the center coordinates and aspect ratios of objects. This shift minimizes the reliance on anchor boxes and enhances both detection speed and accuracy. Collectively, these advancements enable YOLOv8 to preserve the strengths of YOLOv5, while implementing nuanced adjustments and optimizations, thereby improving performance across a variety of scenarios.

### 4.2. MCA Module

The Monte Carlo attention (MCA) module is primarily designed to capture information at different scales, thereby improving the recognition of small objects [29]. Conventional techniques, such as squeeze-and-excitation (SE), produce a 1 × 1 output tensor via global average pooling to adjust inter-channel dependencies; however, they exhibit limitations in leveraging cross-scale correlations. To overcome these challenges, the MCA module creates attention maps of sizes 3 × 3, 2 × 2, and 1 × 1 through randomly sampled pooling operations, which enhances long-range semantic dependencies and effectively mitigates cross-scale issues. The implementation of the Monte Carlo sampling method facilitates the random selection of association probabilities, enabling the extraction of both local information (such as edges and colors) and contextual information (including overall image texture, spatial correlation, and color distribution). This approach significantly enhances the ability to discern the shape and precise location of small objects within images. For input tensor x, the output attention map OA(x) of the MCA module is calculated as follows:

(1) $$OA(x)=\sum_{i=1}^{n}P\left(x,i\right)\cdot f(x,i)$$

where i represents the output size of the attention map, f(x,i) is the average pooling function, and n is the number of randomly generated attention maps. The association probability $P\left(x,i\right)$ satisfies two conditions: (1) $\sum_{i=1}^{n}P\left(x,i\right)=1$; (2) $\prod_{i=1}^{n}P\left(x,i\right)=0$.

### 4.3. DASI Module

In the context of multiple downsampling stages for the detection of small objects, there is a risk that high-dimensional features may lose critical information pertaining to these objects, while low-dimensional features may lack sufficient contextual information. To address this challenge, the dimension-aware selective integration (DASI) method is utilized, which adaptively selects and integrates relevant features based on the size and specific requirements of the target objects [30]. Specifically, DASI aligns high-dimensional and low-dimensional features with the features of the current layer through operations, such as convolution and interpolation. This process involves partitioning the features into four equal segments along the channel dimension, thereby facilitating the effective fusion of features across different scales within the same spatial dimensions. The $h_{i}$, $l_{i}$, and $u_{i}$ represent the i-th (i=1,2,3,4) partition feature of high-dimensional features, low-dimensional features, and the current layer, respectively. These partitions are calculated using the following formulas:

(2) $$\alpha =sigmoid\left(u_{i}\right)$$

(3) $$u_{i}^{\prime }=\alpha l_{i}+\left(1-\alpha \right)h_{i}$$

(4) $$F_{u}^{\prime }=[u_{1}^{\prime },\;u_{2}^{\prime },u_{3}^{\prime },\;u_{4}^{\prime }]$$

(5) $${\hat{F}}_{u}=\delta (Β(Conv(F_{u}^{\prime })))$$

where α is the value obtained by applying the activation function to $u_{i}$; $u_{i}^{\prime }$ is the selective aggregation result of each partition. $F_{u}^{\prime }$ is the result of merging $u_{i}^{\prime }$ along the channel dimension; ${\hat{F}}_{u}$ is the result after convolution (Conv), batch normalization (Β), and rectified linear unit (δ). If α > 0.5, the model prioritizes fine-grained features, and if α < 0.5, it emphasizes contextual features.

### 4.4. MCD-YOLOv8 Model

**Figure 3:**
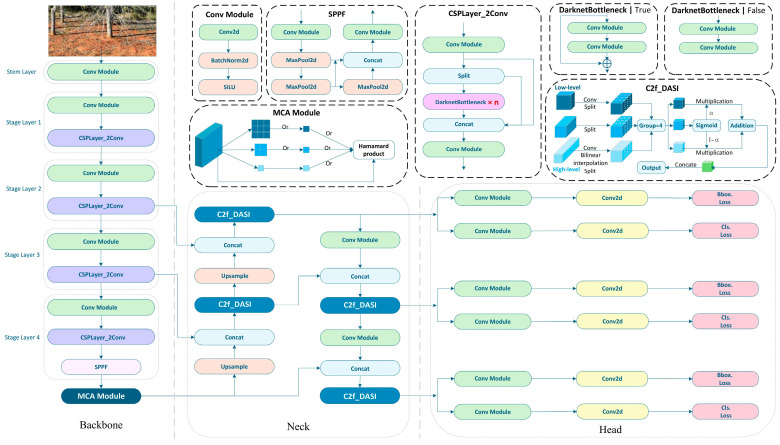
The architecture of MCD-YOLOv8 model.

To facilitate the intelligent identification of the images of soil-dwelling termite damage, this study enhances the YOLOv8 model by developing a multi-objective detection dataset and integrating various modules to tackle challenges associated with the concealment of termite damage, the detection of small targets, and the complexity of image backgrounds. The architecture of the MCD-YOLOv8 model is illustrated in Figure 3. Although the overall framework of MCD-YOLOv8 retains the foundational structure of YOLOv8, consisting of the backbone, neck, and head components, it introduces the MAC module following the SPPF module within the backbone and replaces the C2f module in the neck with the DASI module. The enhancements made to the MCD-YOLOv8 model are particularly notable in two significant aspects:

(1) The integration of the MCA module within the backbone, which facilitates the extraction of both local and contextual information through random sampling, thereby improving the model’s ability to recognize small objects.
(2) The adoption of the DASI module in the neck, which substitutes the C2f module and adaptively enhances fine-grained or contextual features according to varying dimensional requirements, thereby further promoting multi-scale feature fusion.

### 4.5. Model Evaluation Metrics

In order to assess the efficiency and performance of the model, the study employs precision (P), recall (R), average precision (AP), and mean average precision (mAP) as evaluation metrics, utilizing the corresponding formulas, as follows:

(6) $$P=\frac{N_{TP}}{N_{TP}+N_{FP}}\times 100\%$$

(7) $$R=\frac{N_{TP}}{N_{TP}+N_{FN}}\times 100\%$$

(8) $$AP=\int_{0}^{1}P(R)dR$$

(9) $$mAP=\frac{1}{C}\sum_{i=1}^{C}{AP}_{i}$$

where P represents the accuracy of recognition, indicating the proportion of correctly predicted positive samples; R represents the recall rate, indicating the proportion of correctly predicted samples that are actually positive; $N_{TP}$ is the number of true positive samples correctly predicted by the model; $N_{FP}$ is the number of false positive samples incorrectly predicted by the model; $N_{FN}$ is the number of positive samples not predicted by the model; AP is the average precision, representing the area under the P−R curve; a higher AP value indicates better model performance in recognizing specific targets; mAP is the mean average precision, representing the average of AP across all categories; C is the total number of categories.

## 5. Results Analysis

### 5.1. Category Recognition Results

This study develops the MCD-YOLOv8 improved model by combining the MAC and DASI modules based on the YOLOv8 model. The model utilizes original images as input, supplemented by data augmentation techniques to increase the diversity of training samples. During model training, we resized all images to 640 × 640 to maintain consistency and reduce computational load caused by varying original sizes. The backbone part uses four layers of Conv and CSP modules for initial feature extraction, with convolution kernel sizes of 3 × 3 or 1 × 1 and the SiLU activation function. The neck part incorporates FPN and PANet to enhance feature information propagation and introduces a lightweight module. For the loss functions, classification loss primarily uses varifocal loss (VFL), while regression loss combines complete intersection over union (CIOU) and distribution focal loss (DFL). The training process is set for a total of 200 iterations, with a batch size of 16, making it suitable for environments with limited resources and the need for quick data processing. The initial learning rate is set at 0.01; if set too high, it may lead to instability, while too low a rate can slow down convergence.

**Figure 4:**
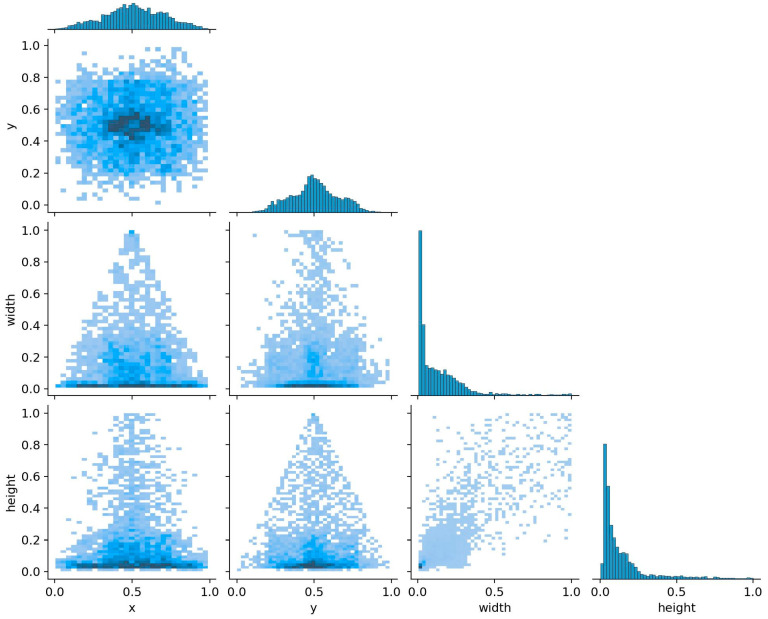
The statistical parameters associated with the labels. The x and y denote the coordinates of the center of the bounding box, with the top-left corner serving as the origin. The width and height parameters indicate the dimensions of the bounding box.

After the process of data augmentation, the dataset has been expanded to include a total of 2096 images, which are classified into the following four distinct categories: deadwood, detector, hole, and mud. The recognition outcomes for these categories, as presented in Table 1, indicate that the enhanced model achieved an overall accuracy of 0.88. Specifically, the deadwood category comprises 1211 instances with an accuracy of 0.891; the detector category includes 131 instances with an accuracy of 0.979; the hole category consists of 125 instances with an accuracy of 0.795; and the mud category contains 2689 instances with an accuracy of 0.857. Furthermore, as illustrated in Figure 4, the centers of the bounding boxes are predominantly located in the central region of the images. The majority of these bounding boxes exhibit widths and heights that are less than one-fifth of the overall image dimensions, with areas that are less than one twenty-fifth of the total image area. It is evident that most soil-dwelling termite activity signs are around 10% in size, classifying them as small targets. Compared to larger- or medium-sized targets, they possess less visual information, making it challenging to extract distinguishing features. Additionally, they are easily affected by environmental factors, making it difficult for detection models to accurately locate and identify these small targets.

**Figure 5:**
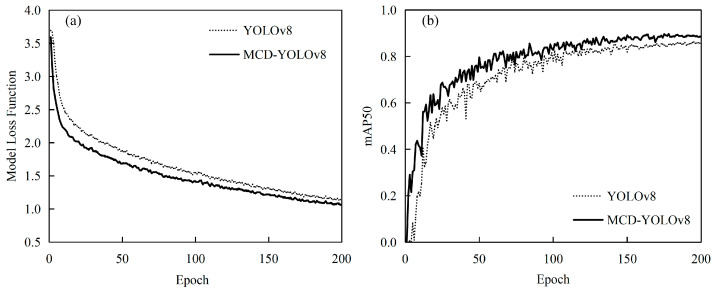
Comparison of loss function curve (**a**) and mAP curve (**b**).

Figure 5 presents a comparative analysis of the loss function curve (Figure 5a) and the mean average precision (mAP) curve (Figure 5b) for the MCD-YOLOv8 and YOLOv8 models. Initially, the loss values for both models exhibit similarities; however, the MCD-YOLOv8 model demonstrates a more rapid and consistent decline in loss values, ultimately achieving a lower stabilization point. Specifically, the loss for MCD-YOLOv8 stabilizes at approximately 1.0, while the YOLOv8 model stabilizes at around 1.2. In terms of the mAP curve, after 50 iterations, the mAP for the MCD-YOLOv8 model increases to approximately 0.8, in contrast to YOLOv8, which reaches only about 0.6 and exhibits greater variability. Ultimately, the mAP for MCD-YOLOv8 stabilizes at approximately 0.886, whereas YOLOv8 stabilizes at 0.862. These findings indicate that MCD-YOLOv8 exhibits enhancements in both recognition accuracy and processing speed, thereby improving its efficiency in practical applications.

### 5.2. Ablation Study

This research enhances the framework for small object detection, addressing challenges related to object occlusion and overlap, through the development of the MCD-YOLOv8 model. To verify the impact of the MCA and DASI modules, an ablation study was conducted based on the YOLOv8 model. In Table 2, “×” indicates “not used”, and “√” indicates “used”. The MCD-YOLOv8 model, incorporating the MCA and DASI modules, shows significant improvements in recognition accuracy and efficiency compared to the original model without any added modules. Compared to YOLOv8, the MCD-YOLOv8 model’s precision increased to 88.0%, up by 6.4 percentage points; the mean average precision (mAP) increased to 88.6%, up by 2.4 percentage points; and the number of parameters decreased to approximately 2.9 million, reducing by 105,320. When using the MCA or DASI module individually, both improve model accuracy, but they affect the parameter count differently. The DASI module effectively reduces model parameters, while the MCA module increases model complexity. Therefore, the MCD-YOLOv8 model, with both MCA and DASI modules, achieves higher recognition accuracy for small objects in complex environments and maintains a lightweight design.

### 5.3. Comparison of Different Model Recognition Effects

To further objectively evaluate the advantages of the MCD-YOLOv8 model, experiments compared the recognition effects of the YOLOv8 model and other improved models. All models were subjected to training utilizing an identical dataset and configuration parameters. Table 3 presents the evaluation metrics with a test dataset for the YOLOv8, NonLocal, PPA, DWR, and MCD-YOLOv8 models. According to the comparative experimental results in Table 3, YOLOv8 has a precision, recall, mAP, and parameter count of 0.816, 0.841, 0.862, and 3,006,428, respectively. Building on YOLOv8, the introduction of the NonLocal, PPA, and DWR modules improves both precision and mAP values but leads to a reduction in the recall rate. Additionally, apart from the DWR module, the other two modules increase the parameter count to some extent. However, the MCD-YOLOv8 model achieves a precision (P) of 0.880, recall (R) of 0.842, and mAP of 0.886, outperforming other models, while having fewer parameters. Thus, the MCD-YOLOv8 model excels in recognition accuracy and lightweight design.

**Figure 6:**
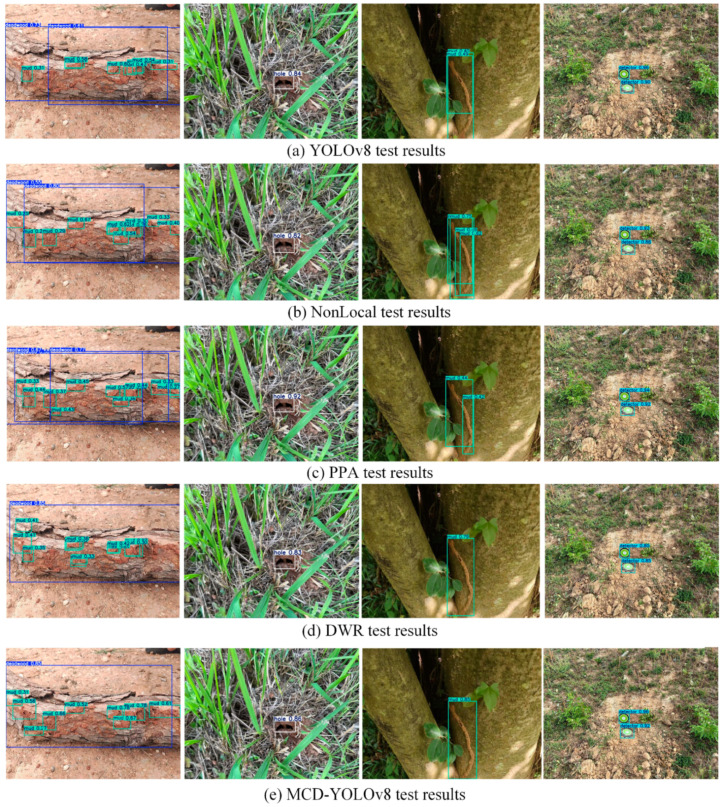
Comparison of recognition results for different models.

To assess the advantages of the enhanced model in detection capabilities, the following four distinct scenarios were evaluated: multi-category detection, small object recognition, partial occlusion, and complex background analysis. Different colored rectangles were utilized to denote the four category labels. The comparative results for the YOLOv8, NonLocal, PPA, DWR, and MCD-YOLOv8 models are illustrated in Figure 6. All models successfully identified the four categories; however, variations in the confidence levels were observed, particularly in the detection of deadwood and mud. The detection confidence for the YOLOv8, NonLocal, PPA, and DWR models ranged from 0.55 to 0.85 for deadwood and from 0.25 to 0.67 for mud. In contrast, the MCD-YOLOv8 model exhibited superior confidence levels, achieving 0.85 for deadwood and a range of 0.31 to 0.78 for mud, thereby facilitating the more accurate identification of soil-dwelling termite activity signs in the majority of instances.

Furthermore, the YOLOv8, NonLocal, and PPA models continue to encounter challenges related to object occlusion and overlap, which hampers their ability to accurately extract the specific features of objects. In contrast, the DWR and MCD-YOLOv8 models demonstrate proficiency in extracting object features and distinguishing occluded objects. However, the DWR model exhibits slightly lower recognition accuracy and precision compared to the MCD-YOLOv8 model. Notably, in the context of detecting small objects (such as mud), the MCD-YOLOv8 model not only effectively differentiates object features but also improves recognition accuracy. Consequently, this model proves to be efficient in isolating targets within a single image and in identifying multiple features of termite damage, demonstrating satisfactory adaptability, stability, and robustness.

## 6. Conclusions

In response to the escalating severity of soil-dwelling termite damage, coupled with the inadequacy of current recognition methodologies to fulfill practical engineering requirements, a novel approach for termite damage identification has been developed based on the YOLOv8 framework. This method incorporates Monte Carlo random sampling and lightweight modules. Specifically, the Monte Carlo attention (MCA) module has been integrated into the backbone to produce attention maps via random sampling pooling operations, thereby addressing cross-scale challenges and enhancing the accuracy of small object recognition. Additionally, a lightweight DASI module has been introduced in the neck to optimize computational efficiency and reduce memory usage, which in turn improves both detection accuracy and processing speed.

When compared to traditional or enhanced models, such as YOLOv8, NonLocal, PPA, and DWR, the MCD-YOLOv8 model demonstrates a reduction in parameter count while preserving adequate feature extraction capabilities and enhancing the accuracy of small object detection. The MCD-YOLOv8 model significantly improves the precision of identifying signs of termite damage in complex environments, minimizes redundant detection boxes, and increases overall detection efficiency. In the context of recognizing termite activity indicators, this model exhibits superior performance, facilitating continuous automated monitoring and markedly enhancing detection efficiency.

In practical applications, particularly for tasks that involve recognizing traces of termite activity under varying lighting conditions and intricate environments, the MCD-YOLOv8 model proficiently and effectively identifies small targets, such as features indicative of termite damage, by mitigating cross-scale effects and employing lightweight module designs. This model presents substantial practical advantages over manual visual inspection methods, which are often characterized by high workloads and low efficiency, thereby providing significant value in termite management. Furthermore, the model holds potential for integration into intelligent inspection technologies, such as drones and robotic dogs [42,49,50], in the future, effectively addressing potential oversights and inaccuracies associated with manual monitoring. This advancement enhances the intelligence of termite activity monitoring and supports effective termite control strategies.

In this study, several advancements in termite damage detection were introduced through the development of the MCD-YOLOv8 model. However, there are notable limitations that warrant further exploration. First, while the model demonstrates improved accuracy in detecting small objects, its performance may still be affected by complex environmental conditions, such as extreme lighting variations or highly cluttered backgrounds. Additionally, the model’s reliance on drone-captured images, although beneficial in terms of large-area coverage, may not always account for the fine-grained details required for precise detection in areas with dense vegetation or structural obstructions. Future work could focus on enhancing the model’s robustness under these challenging conditions, integrating real-time data streams, and incorporating diverse data sources, including sensor-based and acoustic data, to further improve detection reliability. Moreover, exploring the scalability of the model across different geographic regions and varying termite species could broaden its applicability and effectiveness.

## Figures and Tables

**Table 1:** Recognition results for four categories with MCD-YOLOv8 model.

Class	Instances	P	R	mAP50
ALL	4156	0.880	0.842	0.886
Deadwood	1211	0.891	0.884	0.923
Detector	131	0.979	1	0.995
Hole	125	0.795	0.839	0.867
Mud	2689	0.857	0.645	0.758

**Table 2:** Ablation study results.

MCA	DASI	P	R	mAP50	Parameters
×	×	0.816	0.841	0.862	3,006,428
√	×	0.823	0.840	0.860	3,138,012
×	√	0.847	0.829	0.860	2,769,524
√	√	0.880	0.842	0.886	2,901,108

**Table 3:** Comparison of validation results for different models. The bold numbers represent the best values for each parameter of the five models.

Model	Images	Instances	P	R	mAP	Parameters
YOLOv8	418	1142	0.816	0.841	0.862	3,006,428
NonLocal	418	1142	0.840	0.837	**0.886**	3,270,108
PPA	418	1142	0.825	0.817	0.864	5,177,668
DWR	418	1142	0.879	0.838	0.885	2,945,020
MCD-YOLOv8	418	1142	**0.880**	**0.842**	**0.886**	**2,901,108**

## Data Availability

Restrictions apply to the availability of these data. Data were obtained from Hubei Provincial Department of Water Resources and are available from the authors with the permission of Hubei Provincial Department of Water Resources.
